# Surveillance of small, solid pulmonary nodules at digital chest tomosynthesis: data from a cohort of the pilot Swedish CArdioPulmonary bioImage Study (SCAPIS)

**DOI:** 10.1177/0284185120923106

**Published:** 2020-05-21

**Authors:** Carin Meltzer, Erika Fagman, Jenny Vikgren, David Molnar, Eivind Borna, Maral Mirzai Beni, John Brandberg, Bengt Bergman, Magnus Båth, Åse A Johnsson

**Affiliations:** 1 Department of Radiology, Institute of Clinical Sciences, Sahlgrenska Academy at University of Gothenburg, Sweden; 2 Department of Radiology, Division of Radiology and Nuclear Medicine, Oslo University Hospital, Norway; 3 Department of Radiology, Sahlgrenska University Hospital, Gothenburg, Sweden; 4 Krefting Research Centre, Department of Internal Medicine and Clinical Nutrition, Institute of Medicine, Sahlgrenska Academy, University of Gothenburg, Sweden; 5 Department of Medical Physics and Biomedical Engineering, Sahlgrenska University Hospital, Gothenburg, Sweden; 6 Department of Respiratory Medicine, Sahlgrenska University Hospital, Sweden; 7 Department of Respiratory Medicine, Institute of Medicine, Sahlgrenska Academy at University of Gothenburg, Sweden; 8 Department of Radiation Physics, Institute of Clinical Sciences, Sahlgrenska Academy, University of Gothenburg, Gothenburg, Sweden

**Keywords:** Lung, computer applications – detection, diagnosis, radiation safety, digital radiography

## Abstract

**Background:**

Digital tomosynthesis (DTS) might be a low-dose/low-cost alternative to computed tomography (CT).

**Purpose:**

To investigate DTS relative to CT for surveillance of incidental, solid pulmonary nodules.

**Material and Methods:**

Recruited from a population study, 106 participants with indeterminate solid pulmonary nodules on CT underwent surveillance with concurrently performed CT and DTS. Nodule size on DTS was assessed by manual diameter measurements and semi-automatic nodule segmentations were independently performed on CT. Measurement agreement was analyzed according to Bland–Altman with 95% limits of agreement (LoA). Detection of nodule volume change > 25% by DTS in comparison to CT was evaluated with receiver operating characteristics (ROC).

**Results:**

A total of 81 nodules (76%) were assessed as measurable on DTS by two independent observers. Inter- and intra-observer LoA regarding change in average diameter were ± 2 mm. Calculation of relative volume change on DTS resulted in wide inter- and intra-observer LoA in the order of ± 100% and ± 50%. Comparing relative volume change between DTS and CT resulted in LoA of –58% to 67%. The area under the ROC curve regarding the ability of DTS to detect volumetric changes > 25% on CT was 0.58 (95% confidence interval [CI] = 0.40–0.76) and 0.50 (95% CI = 0.35–0.66) for the two observers.

**Conclusion:**

The results of the present study show that measurement variability limits the agreement between DTS and CT regarding nodule size change for small solid nodules.

## Introduction

Since its introduction in 2006, digital tomosynthesis (DTS) of the chest has been undergoing validation for different clinical implications (1–3). DTS can be performed with an upgrade of conventional radiography equipment, where the angular movement of the tube enables separation of overlapping anatomy, with improved visibility of pulmonary lesions compared to chest radiography (4–6). One of the potential roles for DTS is as a low-dose/low-cost alternative to computed tomography (CT) in the imaging of pulmonary nodules, which are defined as parenchymal opacities up to 3 cm in size, with a rounded shape on chest radiograph (CXR) and round or irregular in shape on CT (7).

Incidental nodules are common findings, reported in 14%–50% (8–10) of individuals undergoing chest CT. The majority are benign, although a significant number require follow-up due to the possibility of early stage lung cancer, a decision based on size and risk factors (8,11). Current guidelines from the Fleischner Society (11) recommend surveillance of incidental solid nodules with an estimated risk of lung cancer of ≥ 1%, representing nodules 6–8 mm or 100–250 mm^3^, and the British Thoracic Society (8) recommends follow-up of nodules 5–8 mm or 80–300 mm^3^ in size.

Larger nodules have a greater risk of malignancy and positron emission tomography computed tomography (PET-CT) and biopsy are recommended for further characterization. In the surveillance of nodules, growth is assessed as increase in diameter or volume, or by volume doubling time (VDT). Significant growth is defined as an increase in average diameter ≥ 2 mm or volumetric increase ≥ 25% (8,11). Guidelines from the British Thoracic Society (8), based on the NELSON lung cancer screening study (12), suggest work-up of solid nodules with a VDT < 400 days, surveillance for nodules with a VDT of 400–600 days, and consideration of discontinuation of follow-up for stable/slow-growing nodules (VDT > 600 or < 0 days).

Surveillance of incidental pulmonary nodules detected outside lung cancer screening constitutes an increasing workload for radiology departments. It would be beneficial to have an alternative to CT for nodule surveillance to optimize the use of healthcare resources. The Fleischner Society (11) suggests that CXR may be a low-cost/low-dose alternative to CT for the follow-up of larger nodules that are well visualized and considered low risk. In this situation, DTS would be superior as nodule detection is improved compared to CXR (13), although not on par with CT (14–17). The question has been raised whether DTS could be an alternative to CT regarding surveillance of indeterminate nodules with low risk of malignancy (14,18). Söderman et al. (19) investigated nodule growth on simulated nodules in DTS, and reported that the modality could be applicable, but nodule size, position, and dose level affected the precision of the measurements. Other studies have shown a small difference in size estimates between CT and DTS (20,21). However, to the authors’ knowledge, no published studies have evaluated DTS for surveillance of incidental nodules, which has been recommended (22).

Consequently, the aim of the present study was to investigate the agreement between DTS and CT regarding follow-up of small incidental pulmonary nodules, focusing on change in nodule size.

## Material and Methods

### Participants

**Fig. 1.**
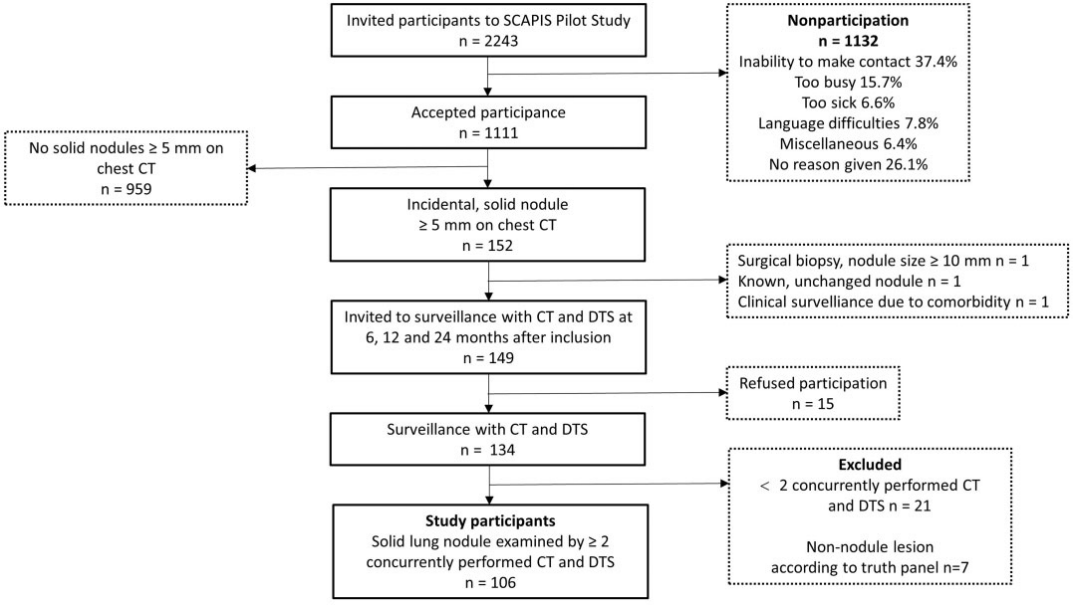
Participant flowchart. CT, computed tomography; DTS, digital tomosynthesis.

The participants were recruited from the prospective, observational pilot Swedish CArdioPulmonary bioImage Study (SCAPIS) (23), which included 1111 randomly invited residents aged 50–64 years who underwent a thorough medical examination, including a chest CT, which revealed 149 cases with incidental pulmonary nodules fulfilling the criteria for surveillance according to regional guidelines based on the Fleischner Society Guidelines from 2005 (24). Participants referred for follow-up of these nodules were invited to the present study, and 90% (n = 134) agreed to participate with written informed consent. The study was approved by the regional Ethical Review Board. A participant flow chart is presented in Fig. 1.

The study protocol included DTS in addition to the scheduled CT at 6, 12, and 24 months after nodule detection. The CT referred to as baseline in the present study is the first follow-up CT with a concurrently performed DTS. In order to facilitate detection of change in nodule size, the two pairs of concurrently performed DTS and CT with the longest time interval were included. Reasons for exclusion were fewer than two DTS performed (n = 21) and participants with lesions that did not meet the criteria for a nodule, decided after a consensus session (n = 7). Finally, a total of 106 individuals were included in the present study. Clinical information, such as smoking exposure and medical history, was based on a written questionnaire. Participant characteristics are presented in Table 1. Non-participants were followed with CT.

**Table 1.** Participant demographics and clinical characteristics.

Gender	
Men	67 (63.2)
Women	39 (36.8)
Age (years)	58.5 (50.3–65.2)
BMI (kg/m^2^)	27.7 (15.7–41.5)
Lung disease as combination of chronic obstructive lung disease/asthma/chronic bronchitis (two or more)	7 (6.6)
Previous cancer	7 (6.6)
2 breast, 1 oral, 1 prostate, 1 appendix, 1 uterine, 1 unknown	
Baseline spirometry	
Normal	76 (71.7)
Pathological	19 (17.9)
Inconclusive	10 (9.4)
No data	1 (0.9)
Smoking status	
Never	40 (37.4)
Active	19 (17.8)
Occasional	2 (1.9)
Previous	46 (43.0)
Pack-years among active and previous smokers	16.5 (0.5–151.9)

Values are given as n (%) or median (range).

BMI, body mass index.

### Image material

#### Computed tomography

CT examinations were performed with a Somatom Definition Flash Dual Source scanner (Siemens Healthineers, Forchheim, Germany) with the following acquisition parameters: constant tube voltage = 120kV; automatic current modulation (CARE Dose4D, 20–25 mAs); and pitch = 0.9–1.2. Images were reconstructed by the B31f kernel with a section thickness of 0.6 mm. The effective dose was estimated by multiplying the dose-length product by a conversion factor of 0.017 mSvGy^−1^ cm^–1^, as recommended by European guidelines (25).

#### Digital tomosynthesis

DTS examinations were performed with the commercially available X-ray system GE Definium 8000, with the VolumeRAD option (GE Healthcare, Chalfont St Giles, UK). Sixty low-dose projection radiographs were acquired during a caudo-cranial tube movement of 30° during a 10-s breath hold at full inspiration, with standard acquisition parameters recommended for DTS (26). The low-dose projections were reconstructed to approximately 60 coronal section images with a reconstruction interval of 5 mm. No retakes were allowed due to radiation considerations. Effective dose for the 70 kg standard patient was estimated by multiplying the dose-area product (DAP) by 0.26 mSvGy^–1^ cm^–2^ (27). DAP was calculated from data in the digital imaging and communications in medicine (DICOM) header of the scout image (28).

#### Nodule size determined by CT

Nodule segmentation was performed using commercially available software (Syngo.via version VB10B_HF03, Siemens Healthineers, Erlangen, Germany), providing estimates of nodule diameters and volume. All analyses were performed by a resident doctor in pulmonology (EB) trained by an expert thoracic radiologist (JV), who also performed an independent analysis of 30 randomly chosen nodules in order to analyze measurement variability for the reference method.

#### Nodule size determined by DTS

##### Study set-up

**Fig. 2.**
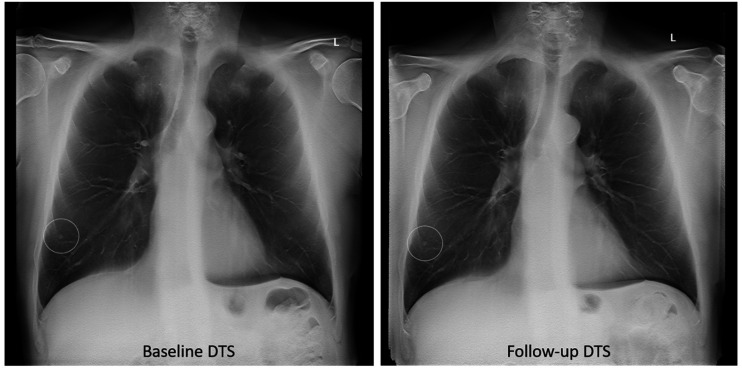
Image example of the appearance of a marked nodule in DTS on baseline and follow-up. Images from baseline and follow-up were displayed in ViewDEX simultaneously for the observers on two flat screens. DTS, digital tomosynthesis.

The largest nodule defined by CT for each participant was marked with a region of interest in the anonymized DTS examinations from baseline and follow-up, including 5–7 images around the focus plane of the nodule. The two DTS were displayed simultaneously, on high-resolution medical-grade flat-panels in the ViewDEX software (29–31), in a room with ambient low light. The study set-up is illustrated in Fig. 2. The cases were presented to the observers in a unique randomized order.

##### Inter- and intra-observer agreement

Two thoracic radiologists; observer 1 (EF) with 16 years and 12 years of experience in radiology and DTS, respectively, and observer 2 (DM) with 10 years and 4 years of experience in radiology and DTS, respectively, independently assessed nodule size on baseline and follow-up DTS. The observers were neither provided with clinical information, nor aware of the time-gap between the examinations.

The observers were familiar with the software and type of study and had access to adjustment of window settings and tools for measurements and zooming. Each DTS was assessed according to: (i) Is the nodule adequately depicted for size measurements? (ii) If yes, measure the maximal long-axis and perpendicular maximal short-axis diameter, given in millimeters with one decimal place.

The diameters from baseline and follow-up were registered in a macro sheet, which calculated a change in volume (%) based on the given measurements. Nodule volume was estimated by the formula of a sphere with a diameter equal to the mean of the two measurements (*a* and *b*), $4\pi \frac{{\left(\frac{a+b}{4}\right)}^{3}}{3}$. To investigate intra-observer agreement, observer 1 reassessed all nodules that were measured during the first session in a new, randomized order six weeks after the first session.

In order to allow the readers the opportunity to judge possible other parameters which might affect apparent nodule size such as the nodule appearing to be located outside of the focus plane, the readers were asked to comment on a qualitative assessment of change in nodule size as characterized as either increased, decreased, or unchanged after completion of their measurements.

### Statistical methods

Statistical analyses were performed using the IBM SPSS Statistics Version 24 software. The mean difference and the 95% confidence interval (CI) were calculated with one sample t-test regarding inter- and intra-observer as well as inter-modality agreement for size estimates. Results were analyzed according to Bland and Altman (32). The spread of the results was estimated by the 95% limits of agreement (LoA), calculated from the mean and SD from the one sample t-test (mean ± 1.96×SD). The cut-off for statistical significance was adjusted for multiple comparisons according to the Bonferroni method (33).

The performance of DTS to detect change in nodule size on CT was analyzed with a receiver operating characteristics (ROC) curve. Positive cases were defined as nodules with volumetric change > 25% on CT (decrease or increase), which was compared to the estimate of volumetric change on DTS. The area under the curve (AUC) describes the probability that a nodule with a volumetric change > 25% on CT will have a higher estimated volumetric change on DTS than a nodule with volumetric change below the threshold. Results were analyzed separately for each observer in order to minimize the effect of clustered data.

## Results

### Examinations and radiation dose

The present study comprises 424 examinations from 106 participants (212 CT and DTS at baseline as well as follow-up) carried out between March 2012 and November 2014. The selection of examinations with the longest time interval resulted in a median follow-up time of 530 days (range = 125–819 days). The estimated median effective dose was 1.6 mSv (range = 0.39–7.01 mSv) for CT and 0.15 mSv (range = 0.09–0.26 mSv) for DTS. The adjusted cut-off for statistically significant *P* values for multiple comparisons was 0.004 (0.05/13).

### Size estimates

#### Estimates of nodule size using CT

Semi-automatic segmentation was possible for all nodules. At baseline CT, the diameter from the segmentation of the 106 nodules was 4–5 mm for 39 (36.8%) nodules, 6–8 mm for 63 (59.4%) nodules, and 9–10 mm for 4 (3.8%) nodules. The corresponding volumes were in the range of 19–277 mm^3^ with 37 nodules ≥ 80 mm^3^ and 23 nodules ≥ 100 mm^3^. The change in average diameter at follow-up was in the range of –1 to 2 mm and the change in relative volume was in the range of –27% to 63%. VDT were > 600 days for 62 nodules and < 0 days for 44 nodules.

The inter-observer analysis agreement resulted in a mean difference of 0 mm^3^ (95% CI = –6 to 7) with LoA in the range of –33 to 33 mm^3^ regarding volume at baseline. The inter-observer agreement for change of nodule size showed a mean difference of 3 mm^3^ (95% CI = –1 to 6) with LoA in the range of –17 mm^3^ to 22 mm^3^, corresponding to LoA for change in relative volume of –29% to 35%.

#### Estimates of nodule size using DTS

**Fig. 3.**
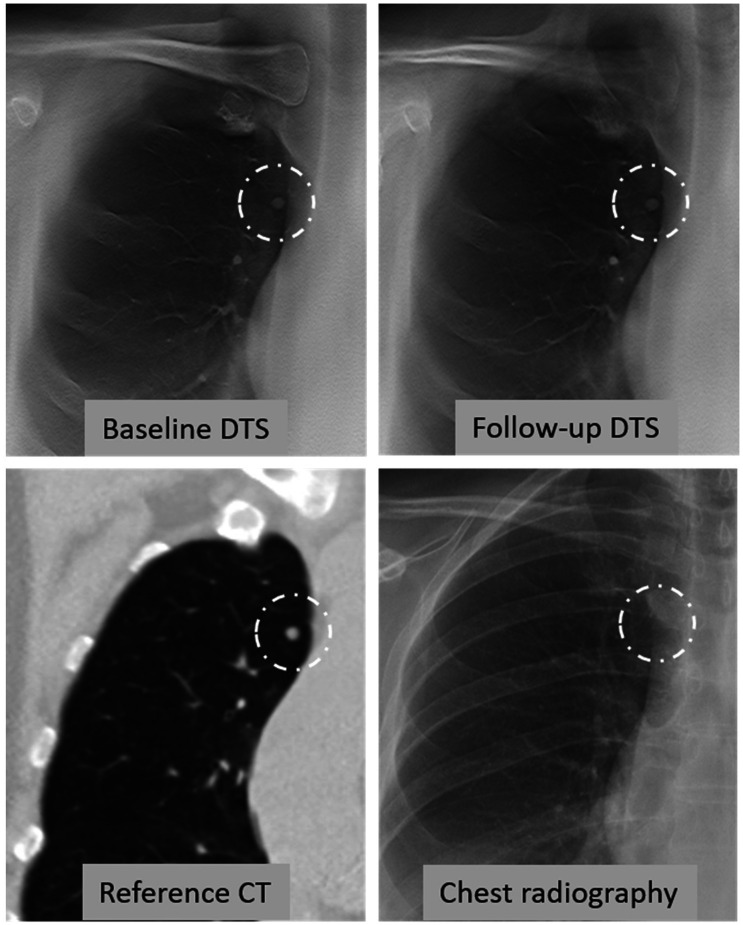
Example of a well-depicted nodule on DTS. A 65-year-old woman, previous smoker (3 pack-years), no history of cancer. Incidental, solid nodule in the right upper lobe with an average diameter of 6 mm on baseline and follow-up CT. Volumetric CT estimates were 66 mm^3^ on baseline, and 87 mm^3^ on follow-up, with a resulting volumetric increase of 32% and VDT of 1300 days. Average diameter based on two diameters on DTS was 5 mm for both baseline and follow-up for both observers, and calculated volume based on the same measurements were 52 mm^3^ and 65 mm^3^ (baseline and follow-up observer 1), and 63 mm^3^ and 71 mm^3^ (baseline and follow-up observer 2), resulting in an volumetric increase of 25% and 13%. VDT was 1500 and 2800 days for observers 1 and 2, respectively. The nodule is not clearly visible on chest radiography. CT, computed tomography; DTS, digital tomosynthesis; VDT, volume doubling time.

**Fig. 4.**
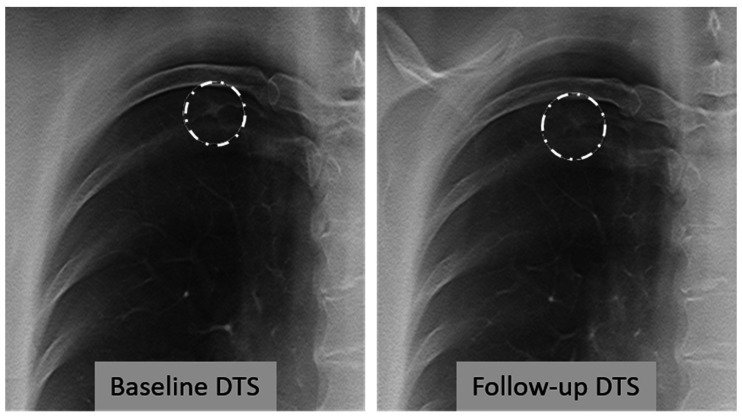
Example of a nodule located outside the focal plane on follow-up. A 59-year-old woman, previous smoker (11.5 pack-years), exposed to cigarette smoke at work, no history of cancer. Incidental, solid nodule in the right upper lobe. Automated measurements on CT provided volume and average diameter of 110 mm^3^ and 7 mm on baseline and 141 mm^3^ and 8 mm on follow-up, and a volumetric change between the examinations of +28% and a VDT of 1632 days. Average diameter base on the two diameters on DTS were 6 mm on baseline for both observers, and 3 and 4 mm on follow-up, and a calculated volumetric change of –76% and –67% for the two observers. The nodule appears to be in the focus plane on baseline DTS, but outside the plane on follow-up. CT, computed tomography; DTS, digital tomosynthesis; VDT, volume doubling time.

**Fig. 5.**
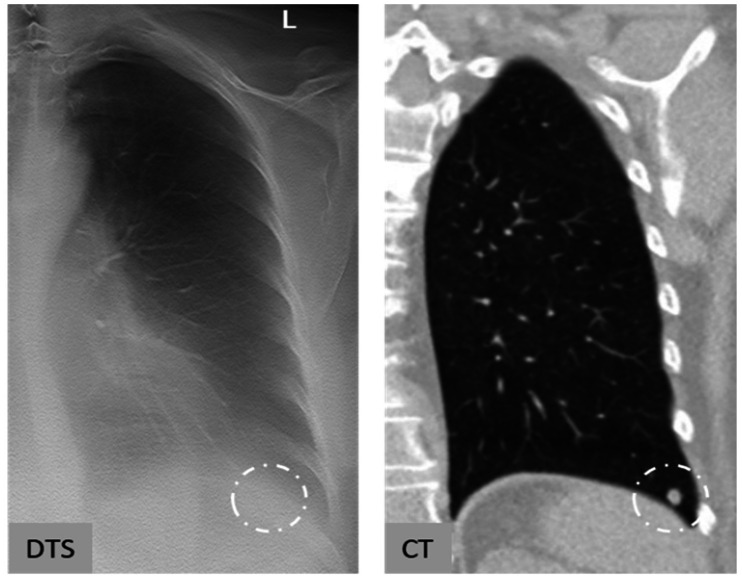
Example of a non-visible nodule on DTS. A 63-year-old man, no history of smoking but exposed to smoke at home, no history of cancer. Incidental, solid nodule with an average diameter of 8 mm in the left lower lobe. Volumetric estimate on CT was 128 mm^3^ on baseline and 208 mm^3^ on follow-up, with a volumetric change between the examinations of +63% and a VDT of 1815 days. The nodule was assessed as non-measurable by both observers on baseline and follow-up DTS. CT, computed tomography; DTS, digital tomosynthesis; VDT, volume doubling time.

A total of 81 (76%) nodules were assessed as measurable by both observers on both baseline and follow-up DTS and these nodules were included for further analyses of measurement agreement. In the second reading, observer 1 judged 79 nodules as measurable on both DTS. Examples of included nodules are given in Figs. 3–5. Regarding nodules ≥ 100 mm^3^, 18/23 (78%) were assessed as measurable on DTS by both observers on both baseline and follow-up. The intra-observer analysis for observer 1 showed a mean difference of –0.1 mm (95% CI = –0.3 to 1.1, LoA = –1.3 to 1.1 mm, *P* = 0.07) at baseline. Inter-observer analysis revealed a mean difference of 0.6 mm (95% CI = 0.4–0.6, LoA = –1.1 to 2.3 mm, *P* < 0.001). There were no statistically significant differences in the intra- and inter-observer analysis regarding change in average nodule diameter, with a mean difference of 0.0 mm (LoA = 1.8–1.9 mm) for inter-observer, and –0.1 mm (LoA = –2.1 to 1.9 mm) for intra-observer agreement. Data are presented in Table 2.

**Table 2.** Inter- and intra-observer agreement regarding average diameter and volume on DTS and inter-observer agreement regarding nodule volume on CT and inter-modality agreement for observer 1.*

	Mean difference (CI)	95% lower LoA	95% upper LoA	P^†^
Nodule diameter DTS inter-observer agreement (n = 81)				
Baseline (mm)	0.6 (0.4–0.8)	–1.1	2.3	< 0.001
Size change (mm)	0.0 (-0.2–0.3)	–1.8	1.9	0.645
Nodule diameter DTS intra-observer agreement (n = 79)				
Baseline (mm)	–0.1 (-0.3–0.0)	–1.3	1.1	0.068
Size change (mm)	–0.1 (-0.4–0.1)	–2.1	1.9	0.230
Nodule volume DTS inter-observer agreement (n = 81)				
Baseline (mm^3^)	18.3 (10.2–26.4)	–53.6	90.1	< 0.001
Size change (mm^3^)	4.3 (2.3–10.9)	–54.4	63.1	0.199
Size change (%)	3.9 (-8.2–16.0)	–103.1	110.9	
Nodule volume DTS intra-observer agreement (n = 79)				
Baseline (mm^3^)	–4.3 (-8.3–-0.2)	–39.5	31.0	0.038
Size change (mm^3^)	0.3 (-3.4–4.1)	–31.6	33.3	0.855
Size change (%)	0.8 (-5–6.7)	–50.3	51.9	
Nodule volume CT measurement variability (n = 30)				
Baseline (mm^3^)	0.4 (-5.9–6.7)	32.6	33.43	0.889
Size change (mm^3^)	2.5 (-1.3–6.2)	–17.4	22.3	0.193
Size change (%)	2.9 (-3.2–8.9)	–28.8	34.5	
Nodule volume on DTS by observer 1 versus CT (n = 81)				
Baseline (mm^3^)	–0.8 (-8.3–6.6)	–66.6	65.0	0.889
Size change (mm^3^)	2.0 (-2.6–6.6)	–38.8	42.7	0.398
Size change (%)	4.5 (-2.5–11.6)	–57.8	66.9	
Nodule diameter on DTS by observer 1 versus CT (n = 81)				
Baseline (mm)	–0.7 (-0.9–-0.5)	–2.5	1.1	0.000
Size change (mm)	–0.05 (-0.2–0.1)	–1.3	1.3	0.520

*Calculations according to Bland–Altman, mean difference, 95% CI, LoA.

^†^Adjusted cut-off for statistically significant *P* values for multiple comparison = 0.004.

CI, confidence interval; CT, computed tomography; DTS, digital tomosynthesis; LoA, limit of agreement.

#### Comparison of nodule size between DTS and CT

**Fig. 6.**
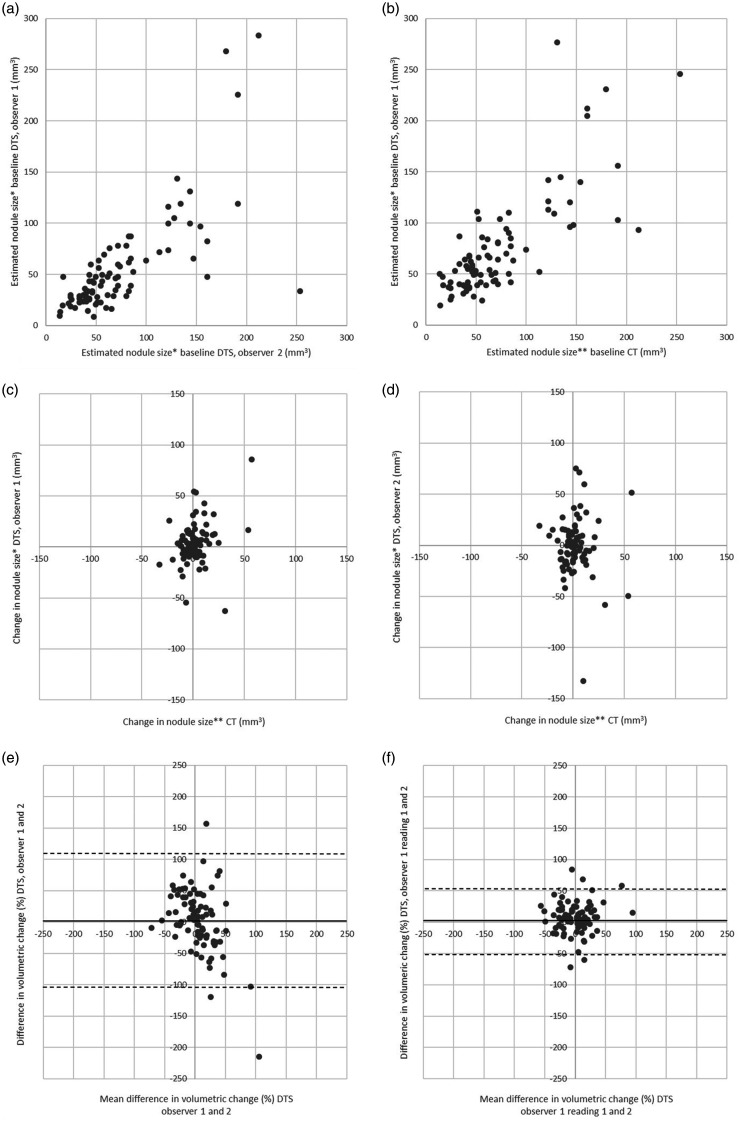
Plots of size estimates on DTS and CT. (a) Scatter plot of nodule volume on DTS for observer 1 (y-axis) and observer 2 (x-axis), n = 81. (b) Scatter plot of nodule volume on DTS (observer 1) (y-axis) and CT (x-axis), n = 81. (c) Scatter plot of change in volume on DTS for observer 1 (y-axis) plotted against change in volume on CT (x-axis), n = 81. (d) Scatter plot of change in volume on DTS for observer 2 (y-axis) plotted against change in volume on CT (x-axis), n = 81. (e) Bland–Altman plot of inter-observer agreement in change of nodule size on DTS, the difference in volumetric change between observers 1 and 2 (y-axis) plotted against the mean difference (x-axis), the mean (black line), and the spread (dotted line) described as the 95% LoA, n = 81. (f) Bland–Altman plot of intra-observer agreement (observer 1), n = 79. CT, computed tomography; DTS, digital tomosynthesis; LoA, limit of agreement*Average diameter based on manual measurements **Semi-automated volumetric estimates.

At baseline DTS, observer 1 measured 85 nodules and observer 2 measured 97 nodules with a mean difference in average diameter compared to CT of –0.7 mm (95% CI = –0.9 to –0.5) and –1.7 mm (95% CI = –2.1 to –1.4) for observers 1 and 2, respectively. The mean difference in change of average diameter between CT and DTS among the 81 nodules measured by both observers at baseline and follow-up was –0.05 mm (95% CI = –0.2 to 0.1, LoA = –1.3 to 1.3 mm) and –0.2 mm (95% CI = –0.3 to 0.02, LoA = –1.8 to 1.4 mm) for observers 1 and 2, respectively. Detailed information on size estimates is given in Table 2 and graphical illustrations of measurement agreement are presented in Fig. 6.

One nodule had a significant change in average diameter on CT according to the 2-mm cut-off recommended by the Fleischner Society (34). This nodule was assessed as unchanged by both manual measurements and visual assessment on DTS by both observers.

The AUC regarding the ability of DTS to detect volumetric changes > 25% reported by CT was 0.58 (95% CI = 0.40–0.76) and 0.50 (95% CI = 0.35–0.66) for observers 1 and 2, respectively. Among 16 nodules with growth > 25% on CT, one and three had a corresponding > 25% increase in volume on DTS reported by observers 1 and 2, respectively. Among nodules ≥ 100 mm^3^ (n = 23), three nodules showed a volumetric growth > 25%, when size and growth was estimated by CT. One of these nodules had a corresponding volume increase > 25% on DTS reported by observer 2.

The qualitative statement regarding estimates of change in nodule size on DTS was compared to change in volumetric estimates on CT, with a 25% cut-off for increase/decrease. The 86 nodules with stable size on CT (change < 25%) was assessed as stable on DTS in 71 (83%) and 48 (56%) participants by observers 1 and 2, respectively.

## Discussion

To our knowledge, the present study is the first that explores the use of DTS for nodule surveillance. Additionally, the study participants were recruited from a unique observational population study and not a lung cancer screening study. The inclusion of participants was based on the 2005 Fleischner Society Guidelines (24) and the results of the present study supports the larger cut-off for nodule surveillance introduced in the 2017 Fleischner Society Guidelines (11) as none of the participants undergoing surveillance developed lung cancer.

The Fleischner Society (11) recommends size estimates based on manual measurements or automated/semi-automated volumetric estimates. Volumetric estimates are more reproducible and thus superior in detection of growth (35–37), but the method is highly dependent on software and reconstruction kernel used, and manual measurements are still often used in clinical practice.

There were no significant differences in diametrical estimates between DTS and CT, and inter- and intra-observer variability in nodule size change showed LoA of ± 2 mm, on par with the 2-mm cut-off for the determination of a significant change in nodule size between follow-up CT examinations (34,38). There are currently no published data on inter-modality agreement regarding surveillance of nodule size between DTS and CT. Previous studies have found comparable size estimates on DTS and CT, but with an inferior agreement between manual measurements on DTS and automated estimates on CT (20,21). Johnsson et al. (20) compared longest nodule diameter at DTS and CT, with a 95% LOA of ± 2.1 mm. Corresponding results for comparison of average diameter in the current study was ± 1 mm, indicating that there was no systematic bias.

The results of the ROC analysis indicate that estimates of change in volume by DTS are close to random guessing in comparison to change in nodule volume as defined by CT. However, this result must be viewed in the light of the patient cohort with small nodules, all with benign VDT. According to the 2017 Fleischner Society Guidelines (11), only 23/106 patients in the present study would have been recommended for surveillance based on nodule volume. Furthermore, the number of nodules exhibiting growth was low (n = 12 for nodules < 100 mm^3^ and n = 4 for nodules > 100 mm^3^ at baseline CT) and we argue that the true positive fraction is too low to adequately address the issue of detecting size change. However, it must be noted that the mean variability in the present study regarding volumetric estimates on CT was 2.9% ± 16.2%, which is in the order of previously studies by Liang et al. (39) and de Hoop et al. (40).

The present study suffers from unknown “true” change in nodule size, issues which can be solved by using simulated nodules, such as the one from Söderman et al. (19), which showed good performance of DTS in detection of nodule growth. However, other factors such as better definition of artificial nodule border, location according to the imaging plane, and fewer motion artefacts are circumstances that impair the ability to draw any direct conclusions to a clinical setting. The present study included all DTS examinations, regardless of image quality, and several nodules that were poorly visible on DTS. Including only high-quality images would probably have influenced measurement agreement, though reducing the generalizability of the results. Depiction in DTS is affected by the location according to the plane of the reconstructed images, illustrated in Fig. 4. Reducing the reconstruction interval from 5 mm to the 1-mm interval often used in breast imaging (41) would increase the likelihood of obtaining a sharp depiction of the nodule in the imaging plane, with a potential for improved measurement precision and inter-observer agreement. However, raw data of images to perform such reconstructions were not available in the present study.

The clinical impact of identifying nodules with moderate growth in a population cohort is unclear; there were no reported malignancies among the 16 participants with nodules with an increase above the threshold of + 25% on CT at a two-year follow-up after the completion of the study. The expected variability on volumetric estimates on CT should be considered in the decision for invasive procedures, and the use of multiple observations and calculation of VDT is valuable when disguising suspicious nodules from those most likely benign (8).

The present study has some limitations. One major limitation is the lack of a reference standard and clinical outcome measurements, though the two-year follow-up revealed no lung cancers. It must be noted that the results from CT are considered as reference standard, meaning that DTS will suffer from the variability in the reference standard, which in the present study exceeded the 25% cut-off for change in volume. A number of participants in the present study underwent unnecessary CT examinations according to the new guidelines from the Fleischner Society (11). However, this is the first study reporting on surveillance of clinical nodules with DTS and the inclusion of the lower limit for nodule surveillance can also be viewed as a scientific strength.

The fact that both observers had prior experience with DTS in clinical practice reduces the ability for generalization of the results of measurement accuracy, but previous studies have reported comparable performance in the detection of nodules using DTS for experienced and inexperienced observers (42,43). The study sample, with small and mostly stable nodules in combination with measurement variability, impairs the ability to assess agreement in change in nodule size. Theoretically, the use of a smaller reconstruction interval of the DTS images may result in improved agreement.

In conclusion, the results of the present study show that measurement variability limits the agreement between DTS and CT regarding the change in nodule size for small solid nodules. Future follow-up studies should investigate whether agreement in size estimates between DTS and CT could be improved by applying a reduction in reconstruction interval, preferably including all relevant nodules sizes.
